# External radicular resorption: Selected cases and review of the literature

**Published:** 2012-06-18

**Authors:** RI Bartok, T Văideanu, B Dimitriu, CM Vârlan, I Suciu, D Podoleanu

**Affiliations:** * Department of Restorative Dentistry, “Carol Davila” University of Medicine and Pharmacy, Bucharest; ** Department of Endodontics, “Carol Davila” University of Medicine and Pharmacy, Bucharest

**Keywords:** external radicular resorption, endodontic treatment, calcium hydroxide, chlorhexidine

## Abstract

External radicular resorption is a pathological process that generates the loss of cementum, dentin and bone, almost irreversibly, involving vital and pulpless teeth. The early stage is asymptomatic and might be diagnosed by a routine radiograph or a clinical examination. Radicular resorption appears because of cementoclastic, dentinoclastic or/and osteoclastic activity. The process of resorption is associated with a damage of the periodontal ligament as a result of injury and necrosis, macrophages are the first cells that are detected, followed by multinucleated cells, odontoclasts, which affect the cementum and dentin.

## Introduction

External radicular resorption is a pathological process that generates the loss of cementum, dentin and bone, almost irreversibly, involving vital and pulpless teeth. The early stage is asymptomatic and might be diagnosed by a routine radiograph or a clinical examination. Radicular resorption appears because of cementoclastic, dentinoclastic or/and osteoclastic activity. There are several types of external root resorption; the most common is the external inflammatory root resorption. The damage of cementum and the infection of the endodontic system may stimulate the osteoclastic activity in periapical tissue and the external root resorption can be initiated. The treatment is related to the stimulation factors (infection or pressure), so, the adequate root canal treatment can almost influence the evolution of the resorptive inflammatory process.

The process of resorption is associated with a damage of the periodontal ligament as a result of injury and necrosis, macrophages are the first cells that are detected, followed by multinucleated cells, odontoclasts, which affect cementum and dentin.

The etiology is multifactorial: trauma, (luxation avulsion replantation), periapical and parodontal inflammation, orthodontic movement, dentoalveolar surgery, periodontal treatment, excessive pressure, occlusal forces, malocclusion, root morphology, chemical irritation (bleaching using hydrogen peroxide 30%), associated systemic diseases, endocrine disorder (Paget’s disease). The etiology requires two phases, injury (mechanical or chemical) and stimulation (by infection or pressure), that act on osteoclasts.

**Figure 1:**
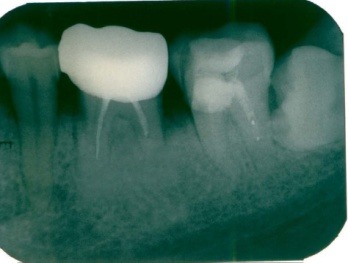
External radicular root resorption with the shortening of the distal root of tooth 36

The denuded mineral tissue is colonized by multinucleated cells and resorption process is initiated (**Fig. 1**).

There might be a genetic component susceptible for the external root resorption that is responsible for the initiation of the resorptive process [**6**]. There is evidence that proinflammatory cytokines play an important role in the pathogenesis of inflammatory root resorption, (Interleukin 1β (IK1β) and tumor necrosis factor alpha (TNFα)). 

Endodontically treated teeth are more susceptible to resorption than normal teeth. 

According to Tronstad (1981), inflammatory resorption might be internal and external; he describes transient, progressive and replacement resorption [**5**].

As far as Andreasen’s classification (1988) is concerned [**1**], there are three types of external resorption: superficial, external resorption with anchylosis and external inflammatory resorption.

In 2009, Hulsmann describes tree types of external radicular resorption: progressive inflammatory, cervical (extracanal invasive resorption) and replacement resorption [**2**].

### Pulpal infection root resorption

**Figure 2:**
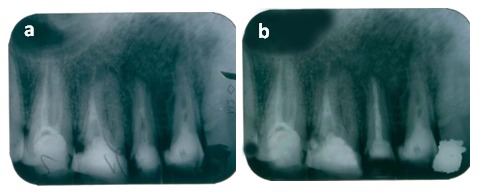
15 external apical root resorption treated with calcium hydroxide and additives (chlorhexidine 2%) for 1 month, (Fig. 2 a), followed by an obturation of the root canal (Fig. 2 b)

**Figure 3:**
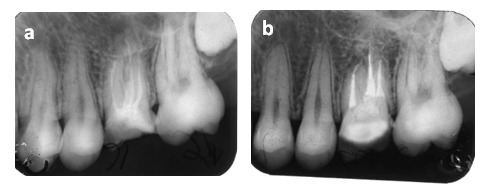
Fig. 3 a Tooth 26 with apical periodontitis and resorptive defect of the palatal root Fig. 3 b Retreatment is indicated and root canal obturation by using adhesive system is performed

The inflammatory external root resorption might be initiated and maintained by pulpo-periapical infection (**Fig. 2 a, b and Fig. 3 a, b**). In addition, we performed a chemomechanical preparation and copious irrigation with Chlorhexidine 2% solution and used Ca(OH)₂ and Chlorhexidine 2% for disinfection as intracanalar dressing, followed by subsequent obturation of the radicular system, using cold lateral condensed gutta-percha

**Figure 4:**
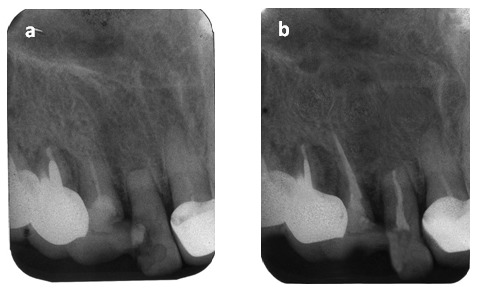
Fig. 4 a 12 external root resorption with the shortening of the root dimension Fig. 4 b Tooth 12 after endodontic treatment)

**Figure 5:**
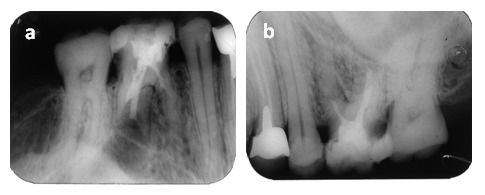
Fig. 5 a 26 external root resorption of the palatal root Fig. 5 b There is no evidence of a resorptive defect progression after a follow-up period of 1 year

The resorptive process of the apical aspect of the root 12 (**Fig. 4 a, b and Fig. 5 a, b**) is probably stimulated by the infectious content of the endodontic system.

### Pressure root resorption

It develops during the eruption of the maxillary canines (that might affect the lateral incisors) or mandibular third molars (that might affect the mandibular second molars) (**Fig. 2 a, b**). Surgery is necessary in order to remove the pressure.

**Figure 6:**
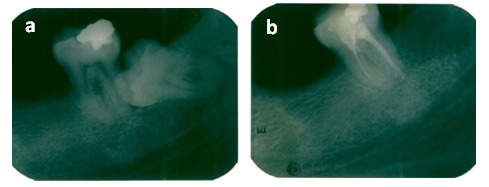
Fig. 6 a Tooth 37 with a lateral root resorption of the apical and coronal segment of the distal root, due to the pressure during eruption of the mandibular third molar Fig. 6 b The odontectomy of 38 is recommended and endodontic treatment of 37 is required, for the limitation of the external resorptive process of the distal root

The treatment of choice for the root resorption related to pressure during orthodontic movement, impacted tooth or tumor requires the removal of the stimulation factor – the pressure.

Orthodontic pressure root resorption is a complication that occurs during the orthodontic movement, the continuous pressure stimulating the resorbing cells in the apical third of the root. Teeth are asymptomatic, with vital pulp in the initial phase. The shortening of the root may occur without radiolucency on the radiograph. Usually, no endodontic treatment is needed, when the orthodontic device is released, the radicular resorption does not continue (it is self-limiting).

### Cervical root resorption

It is a particular form of resorption, probably caused by an infection that originates in the periodontal sulcus, which might stimulate the resorptive process. It is located coronally, to the epithelial attachment, sometimes invading the pulp space. The denuded root surface is populated by resorbing cells. The etiologic factors involved in these invasive forms of resorption might be internal bleaching, trauma, and orthodontic forces.

**Figure 7:**
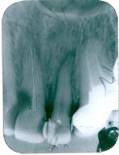
Tooth 21 with cervical radicular resorption lacuna in dentin, expending in coronal and apical direction, with radiolucency of the alveolar bone

The tooth 21 is asymptomatic with cervical root resorption (**Fig. 7**) and it is diagnosed through a routine radiologic exam.

The differential diagnosis may be performed with an internal resorption and subgingival caries.

The adequate treatment requires the removal of the granulation tissue by curettage and the sealing of the resorptive lacuna with restorative materials, in this case, after the endodontic retreatment.

### Replacement resorption (anchylosis)

**Figure 8:**
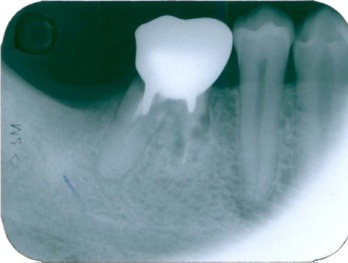
Dentoalveolar anchylosis (46) with “moth-eaten” appearance, and loss of the external root contour of the root

**Figure 9:**
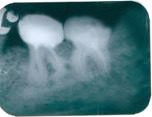
Tooth 36 dentoalveolar anchylosis with an absence of the periodontal space, the resorptive lacunae are filled with bone

It occurs after trauma: luxation or avulsion, which leads to areas of necrosis of the periodontal ligament. Therefore, the osteoclasts are in direct contact with the exposed root surface and the bone is replacing the dentin. So, the bone is attached to the dentin. The periodontal ligament space is missing on radiography (**Fig. 8,9**)

There is no promising treatment for the dento alveolar anchylosis.

In case of avulsion, the most important aspect is the maintenance of the periodontal cells’ viability (correct storage medium and early replantation).

## Discussions

Pathologic resorptions can be radiologically diagnosed only when they have a certain dimension (2 mm diameter, lesions that are placed approximately 1 mm away from the external cortical). 

The most common stimulation factor [**7**] for the inflammatory resorption process is represented by the pulpal infection, [**4**] so, Ca(OH)₂ and chlorhexidine 2% as a vehicle was used as root canal dressing. This intracanalar drug inhibits dentinoclasts by the neutralization of the bacterial lipopolisacharides [**3**] and initiates the reparation process of the resorptive lacunae.

We used chlorhexidine 2% as an irrigation solution and a vehicle for Ca(OH)₂ powder, because of its relative decreased cytotoxic effect and its stable antibacterial property (we did not use sodium hypochlorite because, in case of root resorption, it can easily flow beyond the apex and cause irritation in the periapex). Chlorhexidine increases the pH of dentine, inhibits the osteoclastic activity of acid hydrolase in the periodontal tissue, and, in the same time, it activates the alkaline phosphatase. Chlorhexidine is a long acting antiseptic drug with a remnant effect, which increases the antibacterial effect of Ca(OH)₂.

## Conclusions

• The treatment of inflammatory external root resorption might be predictable, depending on the etiology.

• Pulpal infection may perpetuate the resorptive process, being the most important stimulation factor for the root resorption.

• The non-surgical treatment of the inflammatory resorption has to reduce the bacterial content of the endodontic system and allow the healing of the periradicular space. In addition, an adequate debridement and disinfection - intracanalar medication with calcium hydroxide and additives (chlorhexidine 2%) for 3-6 months was used.

• Subsequent follow up period is needed to correct the estimation about the prognosis of the resorptive process.
